# Molecular characterization and identification of members of the *Anopheles subpictus* complex in Sri Lanka

**DOI:** 10.1186/1475-2875-12-304

**Published:** 2013-08-30

**Authors:** Sinnathamby N Surendran, Devojit K Sarma, Pavilupillai J Jude, Petri Kemppainen, Nadarajah Kanthakumaran, Kanapathy Gajapathy, Lalanthika BS Peiris, Ranjan Ramasamy, Catherine Walton

**Affiliations:** 1 Department of Zoology, Faculty of Science, University of Jaffna, Jaffna 40000, Sri Lanka; 2 Faculty of Life Sciences, University of Manchester, Oxford Road, Manchester M13 9PT, UK; 3 Regional Medical Research Centre, NE region (ICMR), Dibrugarh 786001, Assam, India; 4 Regional Office, Anti Malaria Campaign, Hambantota 82000, Sri Lanka

**Keywords:** *Anopheles subpictus*, *Anopheles sundaicus*, Cytochrome *c* oxidase subunit-I, ITS2, Malaria, Sibling species, Sri Lanka

## Abstract

**Background:**

*Anopheles subpictus sensu lato* is a major malaria vector in South and Southeast Asia. Based initially on polytene chromosome inversion polymorphism, and subsequently on morphological characterization, four sibling species A-D were reported from India. The present study uses molecular methods to further characterize and identify sibling species in Sri Lanka.

**Methods:**

Mosquitoes from Sri Lanka were morphologically identified to species and sequenced for the ribosomal internal transcribed spacer-2 (ITS2) and the mitochondrial cytochrome *c* oxidase subunit-I (COI) genes. These sequences, together with others from GenBank, were used to construct phylogenetic trees and parsimony haplotype networks and to test for genetic population structure.

**Results:**

Both ITS2 and COI sequences revealed two divergent clades indicating that the Subpictus complex in Sri Lanka is composed of two genetically distinct species that correspond to species A and species B from India. Phylogenetic analysis showed that species A and species B do not form a monophyletic clade but instead share genetic similarity with *Anopheles vagus* and *Anopheles sundaicus s.l.,* respectively. An allele specific identification method based on ITS2 variation was developed for the reliable identification of species A and B in Sri Lanka.

**Conclusion:**

Further multidisciplinary studies are needed to establish the species status of all chromosomal forms in the Subpictus complex. This study emphasizes the difficulties in using morphological characters for species identification in *An. subpictus s.l*. in Sri Lanka and demonstrates the utility of an allele specific identification method that can be used to characterize the differential bio-ecological traits of species A and B in Sri Lanka.

## Background

*Anopheles subpictus sensu lato* has a very wide distribution in the Oriental and Australasian regions ranging through Pakistan, India, Sri Lanka, Bangladesh, Myanmar, Thailand, Cambodia, Vietnam, Malaysia, Indonesia, Timor-Leste and Papua New Guinea [1,2]. The taxon exists as a species complex and is a primary vector of malaria in many Southeast (SE) Asian countries and is regarded as a secondary vector in Sri Lanka [2-5]. In India, *An. subpictus s.l.* is reported to exist as a species complex comprising four sibling species *viz*. A, B, C and D [6,7]. Each species is associated with a specific combination of two polytene chromosome inversions *viz*. A = X + ^a^ + ^b^; B = Xab; C = Xa + ^b^; D = X + ^a^b, and stage specific morphometric characteristics such as number of egg ridges, number of branches in the 4 M setae of larvae, and ornamentation of adult palpi [6]. Based on the single inversion (X + ^a^/Xa) on the X chromosome, the presence of species A and B was reported in Sri Lanka [8]. Although no other polytene chromosomal studies looking at the two X chromosome inversions have yet been conducted in Sri Lanka for this taxon, the presence of morphometric characteristics corresponding to the Indian sibling species, led to the reporting of all four (A-D) sibling species from Sri Lanka [5,9,10].

Like any species complex, members of the Subpictus complex show differential bio-ecological traits such as salinity tolerance, susceptibility to common insecticides and vectorial capacity [5,7,11,12]. The development of appropriate vector control strategies when dealing with species complexes, therefore, requires reliable molecular diagnostic tools and an understanding of inter and intraspecific genetic diversity of vector populations [13]. In India, species B is predominant and is a vector in coastal areas of Southern India whereas species A, C and D are predominant in inland areas [1,7] with species A being a vector in West Bengal of India [14]. The members of the Subpictus complex in Sri Lanka also differ in bio-ecological properties [9,11,12] but the full characterization of these requires the development of reliable species diagnostic tools.

The simplest and least expensive way to identify malaria vectors in the field is morphologically. However, there are limitations with using morphological characteristics alone to differentiate sympatric sibling species of anopheline species complexes and closely related taxa [15]. In general, DNA-based methods provide more definitive identification and have an advantage over classical morphological and cytogenetic methods because of their reliability, precision, ease of handling and processing, and their applicability to all mosquito life stages [16]. An excellent species diagnostic molecular marker is the internal transcribed spacer 2 (ITS2) of the ribosomal DNA (rDNA) as its sequence is likely to vary even between closely related species [17].

Morphological descriptions have been given for the four species of the Subpictus complex in India [6]. However, when these diagnostic characters have been applied in Sri Lanka conflicts among putatively diagnostic characters (ornamentation of wing and palpi, number of egg ridges and number of branches of 4 M larval setae) have been observed leading to uncertainty in species identification [18]. Consequently, the present study was designed to determine the taxonomic status of the species complex in Sri Lanka by genetic characterization of the morphologically identified sibling species using ITS2 and the cytochrome *c* oxidase subunit-I (COI) of mitochondrial DNA (mtDNA). The taxonomic designation and ITS2 differentiation among species were subsequently used to develop a DNA based diagnostic assay for the members of the Subpictus complex in Sri Lanka.

## Methods

### Study sites, sample collection and sibling species identification

**Figure 1:**
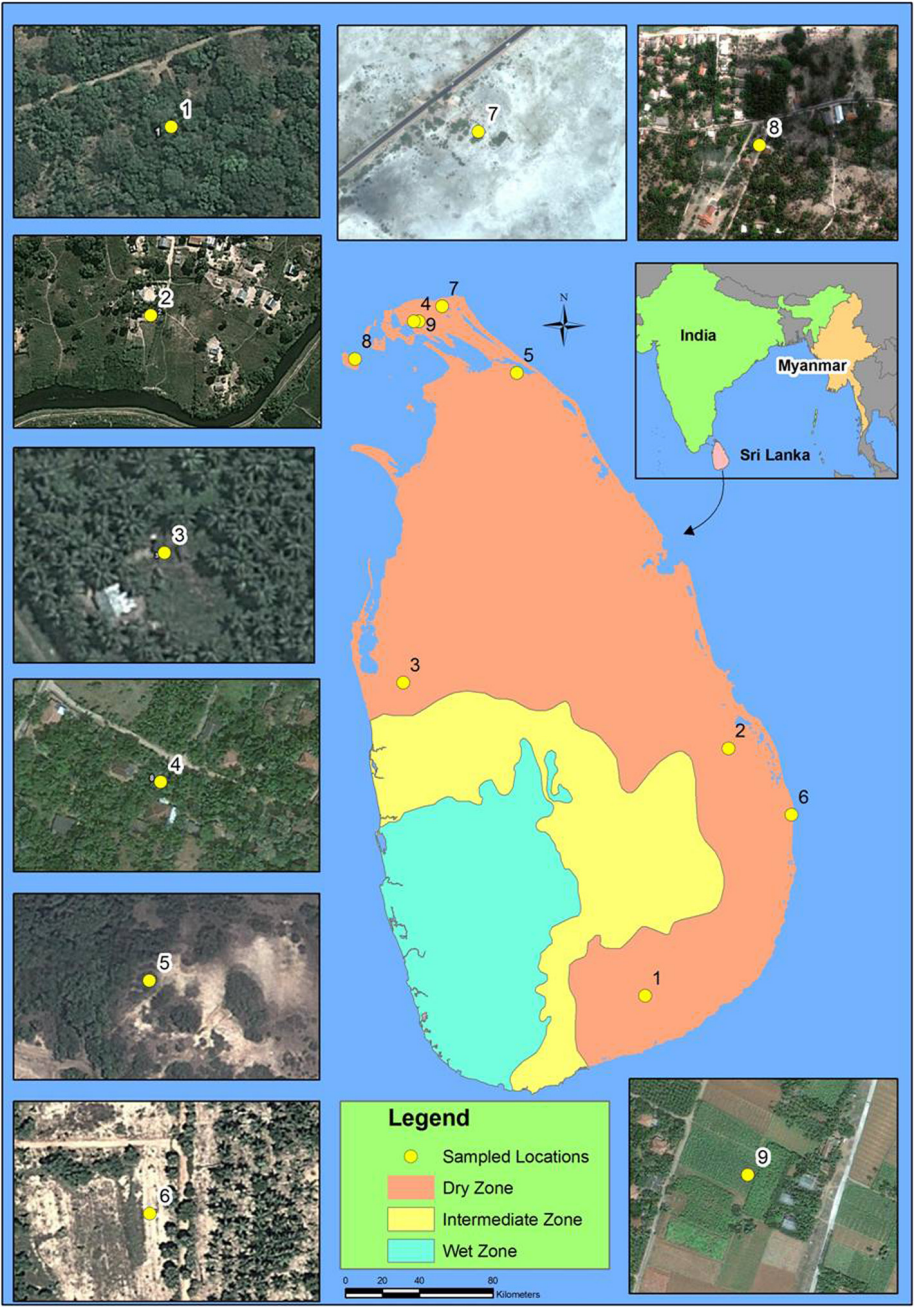
**Map showing the adult and larval collections sites and the climatic zones (dry, intermediate and wet zones classified based on annual rain fall) of Sri Lanka. (1)**. Ranawarunawa, **(2)**. Unnichchai, **(3)**. Thonikkal, **(4)**. Chunnakam, **(5)**. Puliyampokkanai, **(6)**. Kalmunai, **(7)**. Sarasalai, **(8)**. Delft island and **(9)**. Suthumalai.

Mosquito samples (adult and larvae) were collected from nine different localities in Sri Lanka (Additional file 1: Table S1, Figure 1). Adult anopheline mosquitoes were collected during the period of February 2011 to July 2012 using cattle baited hut (CBHC), cattle baited net (CBNC) and hand collection (HC) (using a mouth aspirator) techniques. Larvae were also collected between November 2011 and June 2012 using an 8 cm diameter and 240 ml capacity dipper as described previously [11]. Salinity of water samples was measured using a salinometer (Atago, Japan). The collected adults and larvae were brought to the Zoology Laboratory of the University of Jaffna and identified as *An. subpictus s.l.* using published keys [19-21].

Blood-fed females were maintained individually and single female F_1_ progenies were raised as described previously [11]. The females laying eggs were putatively identified as sibling species A, B, C or D based on the morphological character reported to distinguish these taxa in India, the number of ridges in the egg floats i.e. species A, 31–36; species B, 16–20; species C, 25–29 and species D, 21–24 [6]. Five to ten eggs from each female were placed on a clean microscopic slide and the number of ridges on floats counted under a light microscope (x4, Olympus). Collected larvae were maintained as described previously [11]. Putative sibling species status of the adults that emerged from the collected larvae was determined using published morphometric characteristics (ornamentation of palpi) for the Subpictus complex in India [6]. Any deviation(s) from reported morphological descriptions were noted and the samples were preserved individually in 0.5 ml Eppendorf tubes with silica gel for later molecular characterization.

### DNA extraction and amplification

For DNA extraction, the TENS/phenol-chloroform procedure of Sambrook & Russell [22] was modified by adding the use of Phase Lock Gel (5 Prime) tubes and Qiaex II beads (Qiagen). Phase Lock Gel increases purity and yield of the extracted DNA by forming a tight layer between the aqueous and organic phases while completely trapping the interface. Qiaex II bead suspension, which efficiently binds DNA at pH < 7, was added before ethanol precipitation to minimize the risk of losing the DNA pellet when decanting the ethanol.

Individual mosquitoes were homogenized in 300 μl TENS buffer [22] with 6 μl proteinase-K (10 mg/μl, Merck Chemicals Ltd) and incubated for four hours at 55°C. The homogenate was transferred to pre-spun 2 ml Phase Lock Gel Heavy tubes (5 Prime) and 300 μl of phenol:chloroform:isoamyl alcohol, 25:24:1, pH = 8 (Sigma-Aldrich) was added prior to centrifugation at 13000 rpm for 3 min. After adding 400 μl of chloroform:isoamyl alcohol, 24:1 (Sigma-Aldrich) the tube was centrifuged for 3 min at 13000 rpm. The aqueous phase was transferred to a new tube containing 750 μl of 100% EtOH and 5 μl of Qiaex II suspension (Qiagen). The tube was kept on ice for 10 min and spun at 13000 rpm for 45 min at 4°C. After decanting the EtOH (without disrupting the Qiaex II/DNA pellet at the bottom of the tube), 50 μl of 1:1 mixture of MQ water (Millipore) and QX1 buffer (Qiagen) was added and kept at room temperature for 3 min. Another 150 μl of the same mixture was added and kept for 7 min with gentle mixing every 1–2 min. The tube was centrifuged at 13,000 rpm for 30 sec to pellet the Qiaex II/DNA pellet. Following removal of the supernatant, the Qiaex II/DNA pellet was washed twice with 500 μl of Buffer PE (wash buffer, Qiagen) according to the Qiaex II protocol. The sample was then centrifuged at 13000 rpm for 30 sec and all traces of supernatant removed, after which the pellet was air-dried for 10–20 min. DNA was eluted from the beads by adding 22 μl of Elution Buffer (EB buffer, Qiagen) for 10 min at 50°C. The sample was spun at 13000 rpm for 30 sec and the DNA solution pipetted out. This was repeated again with 20 μl of EB buffer to get a total elution volume of 40 μl (typically 2 μl was retained by the Qiaex II beads). The extracted DNA was refrigerated until further analysis.

The ITS2 region of the rDNA of identified sibling species was amplified using the 5.8S forward and 28S reverse primers [16] and the COI region of the mtDNA was amplified using primers C1-J-1718 and C1-N-2191 [23]. For each amplification, PCR reactions were performed in a 25 μl volume that included 1 μl of DNA, each primer at 0.5 μM, 2.5 mM MgCl_2_, 0.2 mM dNTP mix and 1.25 U *Taq* DNA polymerase in 1x PCR buffer (Bioline, UK). The samples were heated at 94°C for 4 min before 35 cycles of amplification at 94°C for 30 sec, 53°C (ITS2)/57°C (COI), and 72°C for 45 sec followed by a final extension at 72°C for 7 min. The PCR products were purified using GenElute™ PCR Clean-UP Kit (Sigma-Aldrich). Purified PCR products of ITS2 and COI were sequenced in both directions using the Big Dye Terminator V3.1 Cycle Sequencing Kit (Applied Biosystems, USA) on an ABI 3730 automatic sequencer (PE Applied Biosystems, Foster City, CA, USA) at the University of Manchester core sequencing facility. Sequence chromatograms were manually edited in Geneious 4.8.5 [24] and compared with sequence data available in GenBank using BLASTn search.

### Sequence alignment and reconstruction of phylogenetic trees

All generated ITS2 and COI sequences were aligned along with other sequences for *An. subpictus s.l.* and *Anopheles sundaicus s.l.* retrieved from GenBank using ClustalW2in MEGA, version 5 [25]. Phylogenetic relationships among members of the Subpictus complex from Cambodia, India, Myanmar, Sri Lanka, Thailand, and Vietnam along with *An. sundaicus s.l.* from India, Malaysia (Borneo), Myanmar and Timor-Leste were inferred using the maximum likelihood (ML) method. *Anopheles sundaicus* is a species complex and based on known geographical distributions *An. sundaicus* from Borneo is expected to be *An. sundaicus s.s.* and *An.sundaicus* from India is expected to be species D [2] but the species identity of *An. sundaicus* in Myanmar and Timor-Leste is unknown. Therefore, collectively all the samples are referred to as *An. sundaicus s.l*. The substitution model selection was also performed in MEGA5 based on the lowest Bayesian Information Criterion (BIC) value. The Jukes-Cantor model for the ITS2 and Tamura 3-parameter with Gamma distribution model for the COI sequence data set were selected. Bootstrap [26] supports were based on 1000 re-sampled datasets using MEGA, version 5. As *Anopheles vagus* has also been reported to be genetically similar to *An. subpictus s.l.* and *An. sundaicus s.l*. [27], GenBank sequences of *An. vagus* were also included for comparison; ITS2 sequences from China (FJ457631) and India (JN710015) and COI sequences from India (AY834247) and Indonesia (GQ284816).

### Development of an allele specific PCR assay

In order to distinguish sibling species A and B of the Subpictus complex in Sri Lanka an allele specific PCR assay was developed that utilized a common forward primer SubF (5′-3′: ACTGCAGGACACATGAACACCG) and species-specific reverse primers SubA (5′-3′: GCTTGTTGTCGAACCGTGCGAT) and SubB (5′-3′: ATCCGGTTGATACAGGACGCAC). The diagnostic size of the PCR product for species A is ~300 bp while that for species B is ~400 bp. The PCR reactions were performed in 25 μl volumes. Each reaction mix included 1 μl of DNA, each primer at 0.5 μM, 2.5 mM MgCl_2_, 0.2 mM dNTP mix and 1.25 U *Taq* DNApolymerase in 1x PCR buffer (Bioline, UK). The samples were heated at 94°C for 4 min before 30 cycles of amplification at 94°C for 30 sec, 56°C for 30 sec, and 72°C for 30 sec followed by a final extension at 72°C for 7 min. The amplified PCR products were visualized by electrophoresis in 1.5% agarose gels stained with GelRed™ nucleic acid gel stain (Biotium, Inc., USA).

### DNA sequence analysis and population genetic structure based on COI sequences

The COI sequences of Sri Lankan samples along with available sequences for *An. subpictus* of India and Myanmar in the NCBI GenBank were aligned in MEGA, version 5 [25]. Genetic information such as number of haplotypes, segregating sites, haplotype diversity and nucleotide diversity were estimated using DnaSP 5.10 [28].

ModelTest [29] was used to determine the best model of nucleotide substitution. The AIC (hierarchical likelihood tests and Akaike Information Criteria) revealed K81uf (Unequal-frequency Kimura 3-parameter plus Gamma) as the best model (LnL = −1109.652080; AIC = 2231.30416). However, in Arlequin 3.1 [30], the closest model of evolution (Tamura and Nei) was used [31]. Pairwise F_ST_ values were estimated in Arlequin 3.1 [30] and their significance was tested by 1,000 permutations. A statistical parsimony based haplotype network for the populations was created using TCS v1.21 [32].

## Results

### Morphological identification of collected samples

Based on the current morphological descriptions of *An. subpictus* in India [6] all four (A-D) sibling species of the *An. subpictus* complex were found among the samples collected in Sri Lanka. The collection technique, morphometric characters used for identification of the sibling species collected and number of species identified based on morphometric characteristics are given in Additional file 1: Table S1. From across Sri Lanka a total of 887 specimens were processed resulting in 26 individuals identified as species A, 378 as species B, 394 as species C and 89 as species D. Based on these identifications, species A, C and D were predominant in inland localities and species B in coastal localities. Species B larvae were collected from breeding sites with salinity five to 12 parts per 1,000 (ppt). Deviations from the typical taxonomic (morphological) characteristics were observed in the samples identified as species B [18]. For example, the samples collected from coastal areas, namely Delft Island and Sarasali, were identified as species B based on number of egg ridges [6] but had pre-humeral long ornamentation in the wing that is not the taxonomic characteristic of *An. subpictus* but of other closely related species such as *An. sundaicus* and *An. vagus*[19]. Conversely, some specimens identified as species B based on the number of egg ridges [6] and collected from Suthumalai (an inland locality) had larvae with 4 M single setae and pre-humeral long ornamentation in the wing characteristic of the recently reported *Anopheles pseudosundaicus* from South India [33].

### Phylogenetic analysis

**Figure 2:**
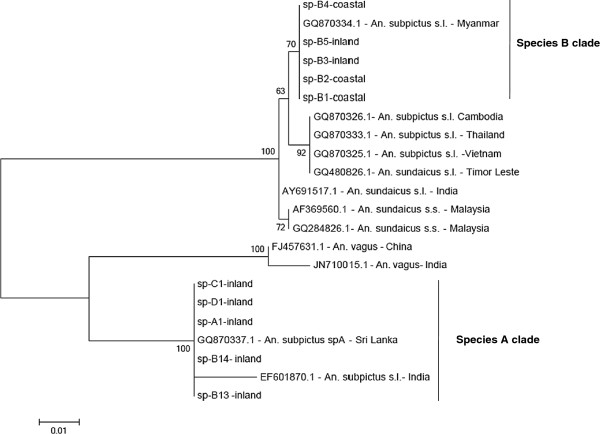
**Phylogenetic analysis based on ITS2 sequences having 387 positions in the data set and constructed using the maximum likelihood method using the Jukes-Cantor model, showing bootstrap values >50%.** Species A clade consists of all specimens belong to sibling species A and species B clade specimens belong to sibling species B. Specimens used for the analysis include morphologically identified species A, B, C and D of Sri Lanka (GenBank accession number: species **A** - KC191825; species **B** – KC191826) and other GenBank entries. The specimen’s GenBank accession number is given along with the country it belongs to in the phylogenetic tree.

All four morphologically identified sibling species were sequenced for the rDNA ITS2 region (n, species A = 2; B = 8; C = 5; D = 2). The resulting 578 bp sequences were aligned with GenBank entries for *An. subpictus* from Cambodia, India, Myanmar, Sri Lanka, Thailand and Vietnam and *An. sundaicus* from India, Malaysia and Timor-Leste and sequences of *An. vagus* from India and China. The sequence dataset used for the final phylogenetic tree reconstruction was 387 bp in length. The resulting tree with sample names and corresponding GenBank accession numbers is given in Figure 2. The phylogenetic analysis revealed that all but two of the specimens from Sri Lanka identified morphologically as species B (including the specimens showing variations in the ornamentation of wing and palpi and number of branches in larval 4 M setae) clustered with GenBank entries for *An. subpictus s.l*. from Myanmar. This clade is referred to as the species B clade and from herein we consider individuals in this clade to belong to species B (see Discussion). However, two of the specimens morphologically identified as species B (based on number of egg ridges) and all other specimens identified as species A, C or D together formed a separate clade along with other GenBank entries of unidentified Indian samples and sequences from Sri Lanka designated as species A. This clade is referred to as the species A clade following earlier notation [34] and from herein individuals in this clade are considered to belong to species A (see Discussion). The corresponding ITS2 sequences for Sri Lanka species A (KC191825) and B (K191826) are deposited in GenBank. The ITS2 gene tree indicates that species A is closely related to *An. vagus* and distinct from species B which falls in a clade with other *An. subpictus* from Southeast Asia and *An. sundaicus s.l*.

**Figure 3:**
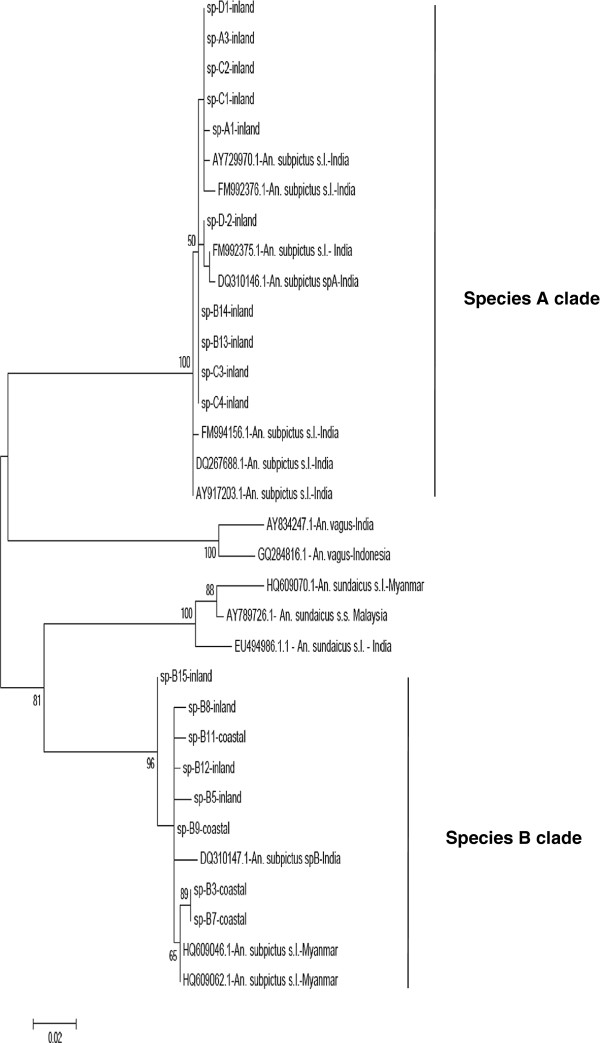
**Phylogenetic analysis based on CO1 sequence having 423 positions in the data set and constructed using the maximum likelihood method using Tamura 3-parameter with gamma distribution, showing bootstrap values >50%.** Species A clade consists of all specimens belong to sibling species A and species B clade specimens belong to sibling species B. The specimen used for analysis include morphologically identified species A, B, C and D of Sri Lanka (GenBank accession numbers: spA3- KC191814; spA1- KC191815; spD2 – KC191816; spC3 – KC191817; spB3 – 191818; spB11 – 191819; sp B9 – KC191820, spB15 – KC191821; spB8 – KC191822, spB12 – KC191823; spB5 – KC191824) and other GenBank entries. The GenBank accession number is given along with the country it belongs to in the phylogenetic tree.

The ~ 500 bp COI sequences obtained for all four morphologically identified sibling species (n, species A = 5; B = 11; C = 5; D = 3) from Sri Lanka were aligned along with GenBank entries for *An. subpictus* from India including specimens identified as species A and B based on egg morphology [35] and Myanmar and *An. sundaicus s.l*. from India, Malaysia and Myanmar. After trimming sequences to the same length, a dataset 423 bp in length was used to reconstruct a phylogenetic tree (Figure 3). In the COI gene tree *An. sundaicus*, *An. vagus*, *An. subpictus* A and *An. subpictus* B each formed distinct clades with similarly deep divergences. In concordance with the ITS2 gene tree, the COI tree suggested that *An. subpictus* A is most closely related to *An. vagus* and *An. subpictus* B to *An. sundiacus* but the bootstrap support is too low to conclude this from this data alone. Despite this, the COI gene tree largely agreed with the ITS2 tree in revealing two distinct clades for *An. subpictus* with individuals falling into the species A or species B clade, entirely consistent with their location in the ITS2 gene tree.

### Genetic diversity and population genetic structure within species A and B

Population genetic structure and genetic diversity was determined for all sequences within species A and within species B, excluding sequences of species B in India which were too few in number to form a separate population. The two populations of species A from Sri Lanka and India had a pairwise *F*_ST_ value of 0.067 (p > 0.05) and the two populations of species B from Sri Lanka and Myanmar had a pairwise *F*_ST_ value of −0.006 (p > 0.05). Given that sample numbers are relatively low it is possible that there is some weak population genetic structure that we have been unable to detect but these results nonetheless indicate that there is very little population substructure within these species over large geographical distances.

**Figure 4:**
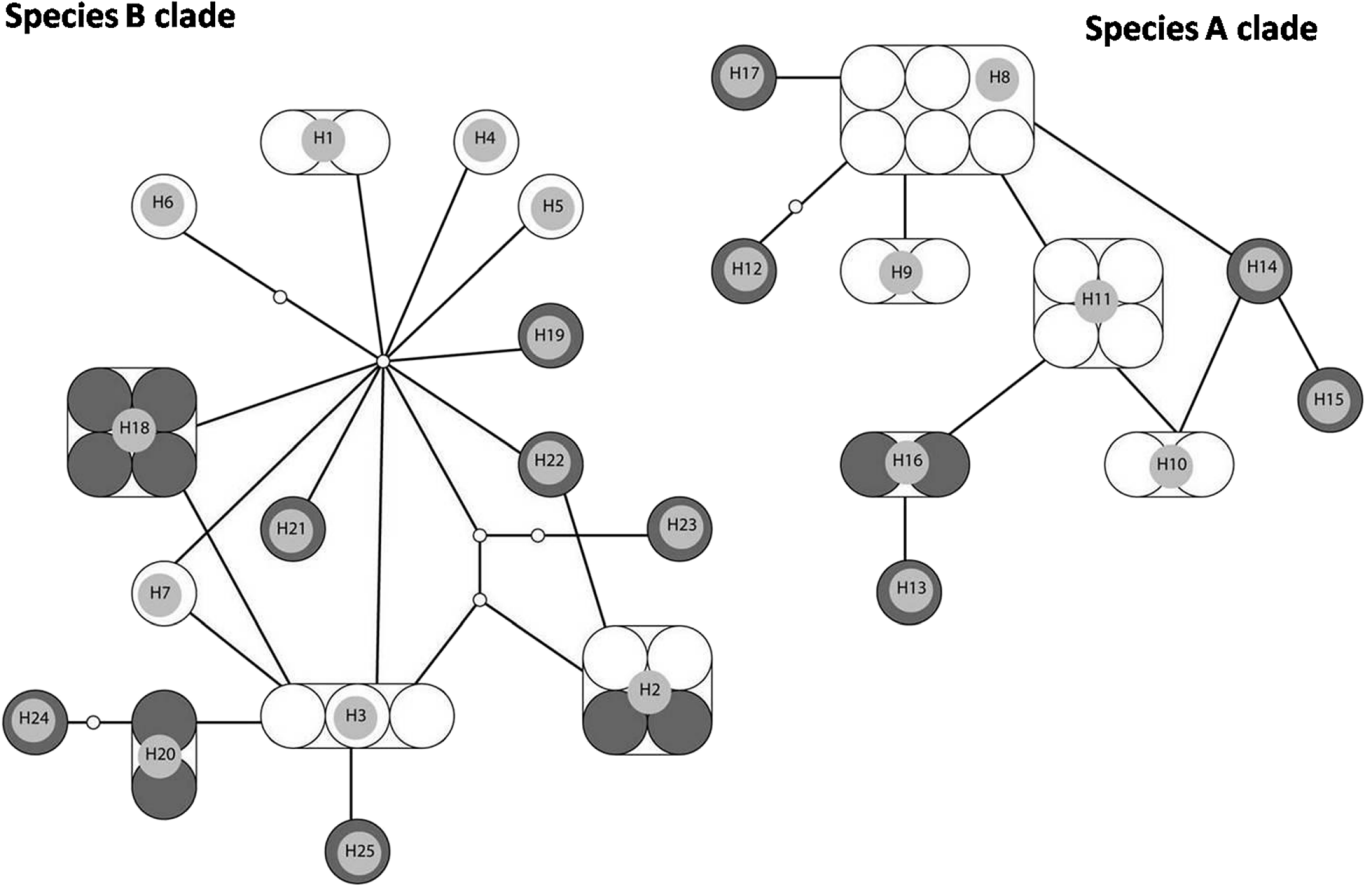
**Haplotype network of COI of the *An. subpictus *populations.** The network clade A is composed of sibling species **A** of Sri Lanka (white large circles) and Indian (black large circles). The network clade B consists of species **B** of Sri Lanka (white large circles) and Myanmar (black large circles). The two networks are separated by 41 mutations. Large circles indicate individual sequences and the haplotype numbers are indicated by H series). Small empty circles represent missing hypothetical haplotypes.

The number of segregating sites, haplotype diversity and genetic diversity estimates for the four populations of species A and B of the Subpictus complex are given in Additional file 2: Table S2. Corresponding haplotype sequences for Sri Lanka species A (KC191814 - KC191817) and B (KC191818 – KC191824) are deposited in GenBank. There were a total of 25 haplotypes. In general, haplotype diversity and genetic diversity are high with the exception of the Sri Lankan population of species A, which has notably lower haplotype and nucleotide diversity. The statistical parsimony haplotype network created for the study populations is given in Figure 4. One haplotype (H2) was shared by populations of species B from Sri Lanka and Myanmar. Conversely, the high frequency (5/25) H8 haplotype was found only in species A from Sri Lanka, underlying the low genetic diversity of this population. Within each of the species, the haplotypes from different countries (Sri Lanka and India in clade A and Sri Lanka and Myanmar in clade B) were intermingled reflecting the lack of genetic population structure as detected by the AMOVA.

### Diagnostic allele specific PCR (AS-PCR) assay for species A and B of the Subpictus complex

**Figure 5:**
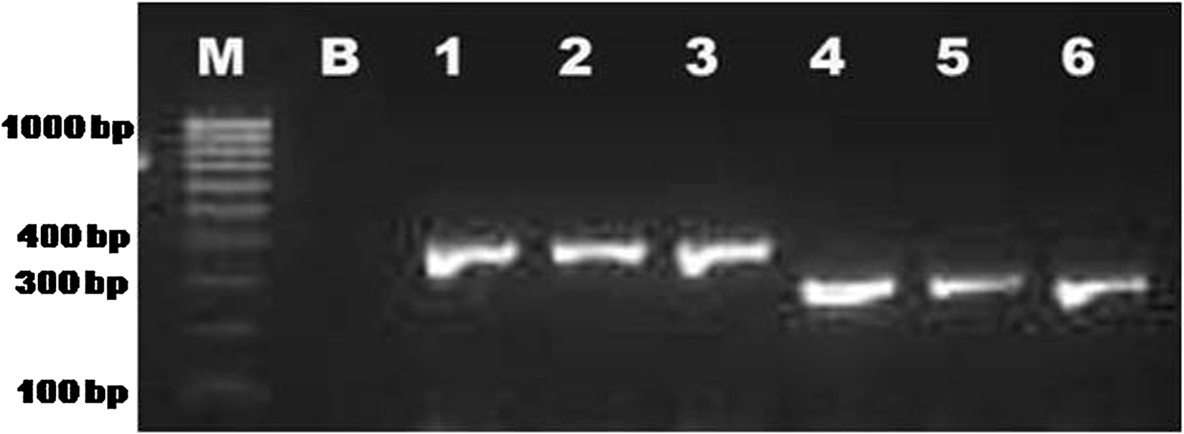
**A 1.5% agarose gel image showing the amplification of the diagnostic fragments for species A and B of the Subpictus complex in the ASPCR assay. M**: 100 bp marker, **B**: control without DNA, **1–3**: species B (B1 – B3); **4–6**: species A (included morphologically identified species A1, C1 and D1).

As the molecular characterization revealed the presence of only two sibling species in Sri Lanka the AS-PCR assay was designed to distinguish only these two members. In the design of species specific primers, fixed base substitutions and indels between the ITS2 sequences were targeted and were mostly positioned at the 3′-extreme end of the primer, where they have the greatest effect on inhibiting extension from mismatched primer-DNA templates. At the same time, consideration was given to generate species-specific amplicons sufficiently different in size to be separated easily on an agarose gel. There is a common forward primer and species-specific reverse primers that were designed to amplify only from *An. subpictus* species A or *An. subpictus* species B and not to *An. vagus* or *An. sundaicus*. A panel of 22 samples collected from different localities and comprising different morphological forms of Subpictus complex in Sri Lanka were used for this assay. The assay clearly separated the specimens into two members of the Subpictus complex. There is no risk of false positives as this assay was unable to amplify products from Sri Lankan samples of *An. vagus* and as *An. sundaicus* is not present in Sri Lanka. All samples were found to produce the expected diagnostic lengths (~300 bp for species A and ~400 bp for species B) in the AS-PCR assay (Figure 5).

## Discussion

The present molecular characterization and population genetic analysis of two loci reveal that members of the Subpictus complex from Sri Lanka fall into two divergent clades. This indicates that the Subpictus complex in Sri Lanka is composed of two genetically distinct species instead of the four sibling species reported previously [5,9]. The populations forming the species A clade in the phylogenetic construction were tentatively designated as *An. subpictus* species A, and the populations that formed the species B clade as *An. subpictus* species B since in the COI gene tree these clades contain specimens from India identified as species A and species B, respectively.

The present study confirms earlier suspicions [15,18] that the morphological-based identification reported for the Indian Subpictus complex [6] is not able to reliably discriminate the members of the Subpictus complex in Sri Lanka. Morphological variation in the ornamentation of palpi and wing has also been reported previously among *An. subpictus* populations in India [36] and Sri Lanka [18]. Several other studies have also found that purported species diagnostic characters were in fact shared across taxa: wing spots in the *Anopheles minimus* complex [37]; spots on wings and proboscis in *An. vagus* and *Anopheles limosus*[38]; and palpi and wing in *An. sundaicus* and *An. subpictus* in Timor-Leste [27]. This demonstrates the difficulties faced in identifying taxa based on morphological characters alone and the utility of molecular methods in identifying and distinguishing closely related *Anopheles* taxa. The diagnostic AS-PCR assay developed here is therefore expected to be very useful for the reliable identification of the two sibling species in Sri Lanka. The lack of population structure seen within species indicates that the method is likely to be widely applicable. The application of this assay in other countries where species A and B are prevalent should be evaluated before use, for example to verify that there is no cross-amplification from any other closely related species present e.g. *An. sundaicus*.

The phylogenetic analysis shows that species A of Sri Lanka and India are genetically divergent from other populations morphologically identified as *An. subpictus* from other South and SE Asian countries including species B in Sri Lanka, India and Myanmar. A previous study from Sri Lanka based on sequences of the ITS2 and D3 domain of rDNA suggested that the majority of the specimens identified morphologically as *An. subpictus* species B in the east coast of Sri Lanka, and some identified elsewhere in SE Asia were genetically close to the well-known salinity-tolerant malaria vector *An. sundaicus s.l.*[15]. This relationship is supported here with both the ITS2 and CO1 markers revealing that *An. subpictus* species A and *An. subpictus* species B do not form a monophyletic clade, as is expected of members of the same species complex, and that they, *An. sundaicus* and *An. vagus* are all relatively closely related. Species A seems to be present only in the Indian subcontinent, mainly in Sri Lanka and India. Species B has a wider distribution than species A being present in Sri Lanka, India and Myanmar. Further studies are required to identify the number of distinct species within the *An. subpictus* complex, their phylogenetic relationships with other closely related species such as *An. sundaicus s.l*. and *An. vagus* and to establish their accurate distributions in South and SE Asia.

A previous study that identified sibling species of the Subpictus complex based on morphological traits revealed that species A, C and D (i.e. species A based on present molecular data) are found inland and breed predominantly in fresh water although they are also reported to breed in slightly brackish water with salinities of up to 4 ppt [11]. However, species B in Sri Lanka can breed in both fresh and brackish water with salinity ranging from 0–30 ppt [11]. In addition, the predominantly inland species (morphologically identified as C and D and inferred from molecular data herein to be species A) are more resistant to common insecticides than the coastal population of species B [12]. This differential adaptation is likely caused by higher selection pressure on species A as decades of insecticide application to control malaria and for use in agriculture has mainly targeted the freshwater-inland areas in Sri Lanka. Population bottlenecks that arise due to insecticide application may explain the lower haplotype and nucleotide diversity of species A in Sri Lanka. Insecticide selection pressure has been reported as a factor underlying the pattern of genetic diversity in other mosquito species such as *Aedes aegypti*[39,40].

The prevalence of species B at both inland and coastal localities indicates that it is not appropriate to classify populations of the Subpictus complex as strictly inland or coastal [41] especially in the Sri Lankan context. While the origin and spread of species B is yet to be established it should be noted that species B of Sri Lanka shared a haplotype with *An. subpictus* populations of Myanmar and that there was a lack of genetic differentiation within species B even across the large geographical distance from Sri Lanka to Myanmar. This lack of intraspecific differentiation could reflect a demographic history of these populations in which they have been derived recently and so have not accumulated genetic differences [17]. The ability of species B to flourish in brackish water may have facilitated the dispersal between Sri Lanka and Myanmar along a predominantly coastal route.

The molecular studies reported here concur with karyotypic studies which to date report only species A and B from Sri Lanka based on a single inversion in the X-arm [8]. It would be of great interest to determine if this inversion difference corresponds to the molecular types of the ITS2 and COI markers as it is predicted from this study. The present study also showed that the molecular form of species A in Sri Lanka encompassed the full range of variation in number of egg ridges reported in species A, C and D in India. However, the possibility that additional species (or distinct chromosomal forms) of the *An. subpictus* complex exist in Sri Lanka, as reported in India [6], which cannot be detected by the molecular markers used here cannot be precluded. Although the loci used here, particularly ITS2, are generally able to detect very closely related species they can prove ineffective where speciation has been very recent [42] or is perhaps even ongoing, as in the S and M molecular forms of *Anopheles gambiae*[43,44]. To fully determine if there are species (or chromosomal forms) in the Subpictus complex in addition to species A and B described here, a coordinated study of polytene chromosomes, molecular markers, morphological characters and ecological characters would be required, ideally in both Sri Lanka and India.

## Conclusion

Sri Lanka has now entered the pre-elimination phase of malaria with a low number of reported cases in recent times but the continuous monitoring of vector populations to identify the potential for malaria transmission and optimal vector control strategies is essential to prevent future outbreaks [45]. The presence of two or more uncharacterized sibling species of a species complex in a particular locality can conceal real disease transmission patterns and lead to sub-optimal vector control programmes. The present molecular characterization of the Subpictus complex in Sri Lanka shows the existence of two distinct sibling species namely species A and B and reports a DNA based diagnostic technique to distinguish them in Sri Lanka. Studies in coastal areas in the North Central Province [9] and inland areas of North Central and Eastern Provinces [3,4] of Sri Lanka indicate the involvement of *An. subpictus* in malaria transmission. These are therefore key areas for the application of this diagnostic AS-PCR assay to study species biology relevant to vector control and for vector incrimination studies.

## Competing interests

The authors declare that they have no competing interest.

## Authors’ contribution

SNS, CW and RR designed the study. PJJ, KG, NK and LBSP did field collections and identification. PK developed the modifications in the DNA extraction protocol. SNS did laboratory studies. SNS, DKS and PK did data analysis. SNS, CW and RR wrote the manuscript. DKS and PK contributed to the content of the manuscript. All authors read and approved the final manuscript.

## Supplementary Material

Additional file 1: Table S1 Sample collection sites and distribution of morphologically identified sibling species from Sri Lanka. Click here for file

Additional file 2: Table S2 Summary of the genetic diversity estimate for *An. subpictus* population of Sri Lankan (SLK), India (IND) and Myanmar (MYN) based on CO1 sequence. Click here for file
