# Supplementary material for: Molecular characterization and identification of members of the Anopheles subpictus complex in Sri Lanka
Source: Malar J. 2013 Aug 30;12:304. doi: 10.1186/1475-2875-12-304 (PMC3766661; doi:10.1186/1475-2875-12-304)
Supplement: Additional file 1: Table S1 — Sample collection sites and distribution of morphologically identified sibling species from Sri Lanka. [file 1475-2875-12-304-S1.doc]

**Additional File 1.** Sample collection sites and distribution of morphologically identified sibling species from Sri Lanka.

| **District** | **Locality** | **Habitat type** | **Latitude: Longitude** | **Identification character** | **^*^Collection technique** | **Number of individuals identified per putative sibling species** | | | | **Salinity of breeding site (ppt)** |
| --- | --- | --- | --- | --- | --- | --- | --- | --- | --- | --- |
|  |  |  |  |  |  | **A** | **B** | **C** | **D** |  |
| Ampara | Kalmunai | Coastal | 7^0^17'39.85" N  81^0^51'38.00" E | Egg | CBNC |  | 78 | 11 |  |  |
| Batticaloa | Unnichchai | Inland | 7^0^37'10.66" N  81^0^33'04.79" E | Egg | CBHC |  |  | 35 | 23 |  |
|  |  |  |  | Palpi | CBNC |  |  | 6 |  |  |
| Hambantota | Ranawarunawa | Inland | 6^0^24'27.48" N  81^0^08'25.50" E | Egg | CBNC |  |  | 10 |  |  |
|  |  |  |  | Palpi | CBNC |  | 90 |  |  |  |
| Jaffna | Suthumalai | Inland | 9^0^42'47.56" N  80^0^00'08.01" E | Egg | HC | 7 |  |  |  |  |
|  |  |  |  | Egg | CBNC |  | 24 | 115 |  |  |
|  |  |  | 9^0^42'47.56" N  80^0^00'08.01" E | Egg | CBNC |  |  |  | 14 |  |
|  | Sarasali | Marshy | 9^0^47'14.23" N  80^0^08'29.09" E | Palpi | LC |  | 25 |  |  | 5-10 |
|  | Delft Island | Coastal | 9^0^31'38.36" N  79^0^42'43.33" E | Palpi | LC |  | 65 |  |  | 10-12 |
|  | Chunnakam | Inland | 9^0^44'58.47" N  80^0^00'08.01" E | Egg | CBNC |  | 15 |  |  |  |
|  |  |  |  | Palpi | CBNC |  |  | 28 |  |  |
|  |  |  |  | Palpi | LC | 12 |  | 137 | 34 | 0 |
| Kilinochchi | Puliyampokkana | Marshy | 9^0^27'39.49" N  80^0^30'38.57" E | Egg | CBNC |  | 81 |  |  |  |
| Puttalam | Thonikkal | Inland | 7^0^56'31.06" N  79^0^57'13.03" E | Egg | CBHC | 7 |  |  | 10 |  |
|  |  |  |  |  | CBNC |  |  |  | 8 |  |

^*^Collection techniques: CBNC- cattle baited net collection; CBHC- cattle baited hut collection; HC- hand collection; LC- larval collection. The locations of the different sites are shown in Figure 1
