# Supplementary material for: Molecular characterization and identification of members of the Anopheles subpictus complex in Sri Lanka
Source: Malar J. 2013 Aug 30;12:304. doi: 10.1186/1475-2875-12-304 (PMC3766661; doi:10.1186/1475-2875-12-304)
Supplement: Additional file 2: Table S2 — Summary of the genetic diversity estimate for An. subpictus population of Sri Lankan (SLK), India (IND) and Myanmar (MYN) based on CO1 sequence. [file 1475-2875-12-304-S2.doc]

**Additional File 2:** Summary of the genetic diversity estimate for *An. subpictus* population of Sri Lankan (SLK), India (IND) and Myanmar (MYN) based on CO1 sequences.

| **Species** | **No. of samples** | **No.of segregating**  **sites** | **No. of haplotypes** | **^*^Haplotype frequency** | **Haplotype diversity (Hd)** | **Nucleotide diversity**  **±s.d** |
| --- | --- | --- | --- | --- | --- | --- |
| SubA SLK | 13 | 3 | 4 | H8 (5); H9 (2); H10 (2); H11 (4) | 0.769 | 0.0027  **±** 0.0021 |
| SubB SLK | 11 | 13 | 7 | H1 (2); **H2** (4);  H3 (3); H4 (1); H5(1); H6(1); H7(1) | 0.909 | 0.0082  **±** 0.0051 |
| SubA IND | 7 | 8 | 6 | H12 (1); H13 (1); H14 (1); H15 (1); H16 (2); H17 (1) | 0.952 | 0.0075  **±** 0.0051 |
| SubB  MYN | 14 | 12 | 9 | **H2** (4); H18 (4); H19 (1); H20 (2); H21 (1); H22 (1); H23 (1); H24 (1); H25 (1) | 0.912 | 0.0080  **±** 0.0049 |

^*^Haplotypes in bold indicates theoccurrence of the haplotype in more than one population and the number in parenthesis indicates the frequency of the haplotype.

Levels of significance: **p*= <0.05; ***p*= <0.01, ****p*=< 0.001
